# Body Mass Index as a Predictor of COVID-19 Severity in ICU Patients in Saudi Arabia: A Retrospective Analysis

**DOI:** 10.7759/cureus.52470

**Published:** 2024-01-17

**Authors:** Abdulrahman A Alomran, Khalid A Althubaiti, Hussain A Alabdullah, Heba B Al Bisher, Atheer Awadh, Hind A Al Shankiti, Laura Almazyad, Leen A Aljandul, Abrar T Aljohani, Obay W Dhafar, Zeyad A Alzahrani, Ayman M Kharaba

**Affiliations:** 1 Surgery, King Saud University, Riyadh, SAU; 2 Surgery, Ibn Sina National College for Medical Sciences, Jeddah, SAU; 3 College of Medicine, Imam Abdulrahman Bin Faisal University, Dammam, SAU; 4 College of Medicine and Medical Science, Arabian Gulf University, Manama, BHR; 5 College of Medicine, Taif University, Taif , SAU; 6 Emergency Medicine, Al Qunfudah General Hospital, Al Qunfudah, SAU; 7 College of Medicine, King Saud bin Abdulaziz University for Health Sciences, Riyadh, SAU; 8 College of Medicine, Imam Mohammad Ibn Saud Islamic University, Riyadh, SAU; 9 Family Medicine, Faculty of Medicine, Ibn Sina National College for Medical Sciences, Jeddah, SAU; 10 Internal Medicine, King Abdulaziz University Hospital, Jeddah, SAU; 11 Internal Medicine, Presidency of State Security, Saudi Arabia, SAU; 12 Internal Medicine Intensive Critical Care Unit, King Fahad Hospital, Madinah, SAU

**Keywords:** icu patient study, electronic health records, retrospective cohort design, saudi arabian context, bmi impact analysis, covid-19 severity

## Abstract

Introduction

The global coronavirus disease 2019 (COVID-19) pandemic has prompted research into various risk factors, including the role of body mass index (BMI) in disease severity. This study specifically examines the correlation between BMI and the severity of COVID-19 among intensive care unit (ICU) patients in Saudi Arabia, addressing a gap in region-specific data. The study aims to assess the impact of BMI on the severity of COVID-19 in a Saudi Arabian ICU patient cohort, providing insights into how this relationship varies in different demographic contexts.

Materials and methods

Employing a retrospective cohort design, the study analyzed data from adult ICU patients in Saudi Arabia diagnosed with COVID-19. It focused on variables like BMI at admission, demographic information, and COVID-19 outcomes including severity, recovery, and mortality. Statistical analysis involved regression models, adjusting for age, gender, and comorbidities.

Results

Unlike global observations, the study found no significant correlation between BMI and COVID-19 severity in the Saudi Arabian context. This suggests that in this specific demographic, other factors may be more critical in determining the severity of the disease.

Conclusion

Our findings challenge the global consensus on BMI as a key factor in COVID-19 severity, highlighting the importance of regional differences in disease dynamics. They underscore the need for localized healthcare strategies and further research into diverse demographic factors affecting COVID-19. This study contributes to a broader understanding of the pandemic and encourages region-specific approaches in both clinical and public health spheres.

## Introduction

The ongoing coronavirus disease 2019 (COVID-19) pandemic has posed unprecedented challenges to global health systems, revealing critical associations between various health factors and the severity of the virus diseases. Among these, the role of body mass index (BMI) in predicting the severity of COVID-19, especially in intensive care unit (ICU) patients, has emerged as a significant area of research [1]. This introduction aims to set the stage for understanding the essence of this research area, its critical importance in the current medical landscape, and the specific objectives guiding our investigation.

Recent studies have consistently shown a link between higher BMI and increased severity of COVID-19 complications, particularly in patients admitted to ICUs [2,3]. This correlation is critical in regions like Saudi Arabia, where varying BMI levels are prevalent and can significantly impact COVID-19 management strategies [4]. Understanding this relationship is not only crucial for patient treatment but also for public health policies, especially considering disparities in obesity and ethnicity/race impacts on COVID-19 outcomes [5].

The scientific background of this investigation is rooted in the growing evidence suggesting that patients with elevated BMI levels are at a heightened risk of severe outcomes when infected with COVID-19. This includes increased likelihood of hospitalization, the need for mechanical ventilation, and even mortality [6,7]. The rationale for focusing on Saudi Arabian ICU settings stems from the region's unique demographic and health profiles, which offer valuable insights into the broader implications of BMI on COVID-19 severity [8].

Despite the established links between high BMI and adverse COVID-19 outcomes, a comprehensive understanding of this association, especially in specific geographical contexts like Saudi Arabia, remains underexplored. This study addresses the central research question: How does BMI influence the severity of COVID-19 among ICU patients in Saudi Arabia? This question is bolstered by evidence indicating significant variability in COVID-19 impacts across different BMI categories [9,10].

The central objective of this study is a comprehensive retrospective analysis to elucidate the relationship between (BMI) and the severity of COVID-19 in ICU admissions in Saudi Arabia. This endeavor is tailored to be specific, measurable, achievable, relevant, and time-bound, focusing exclusively on Saudi Arabian ICU settings. The analysis will explore three key aspects: firstly, assessing the correlation between various BMI categories and the severity of COVID-19, aiming to understand the extent to which BMI influences clinical outcomes. Secondly, it will delve into the implications of these findings for clinical management and public health strategies within Saudi Arabia [11,12], providing actionable insights for healthcare professionals and policymakers. Lastly, the study aims to identify and analyze any potential disparities in COVID-19 outcomes based on BMI within the Saudi population [13], contributing to a more nuanced understanding of how demographic factors impact the severity of the disease. This comprehensive approach is designed to not only enhance clinical decision-making but also to inform public health strategies in a region-specific context, aligning with the ongoing global efforts to combat the pandemic effectively.

This research is timely and necessary, providing critical insights into how BMI impacts COVID-19 severity in a specific regional context. The findings are expected to indicate key areas for intervention, offer implications for managing ICU patients with varying BMI levels, and contribute to the broader understanding of COVID-19's impact across different populations. Moreover, this study will advance academic knowledge and practical understanding of the interplay between obesity and infectious diseases like COVID-19 [14,15].

This study aims to comprehensively analyze the association between BMI and COVID-19 severity in ICU settings in Saudi Arabia. By focusing on this specific aspect, the research aims to contribute to the global understanding of COVID-19, offering crucial insights for healthcare professionals and policymakers. The expected outcomes include a better understanding of the risk factors associated with severe COVID-19 cases and enhanced strategies for managing patients with varying BMI levels in ICUs [16].

## Materials and methods

Study design

This study employed a retrospective cohort design, specifically chosen for its ability to analyze pre-existing data over a period, thereby facilitating the understanding of how BMI influences COVID-19 outcomes in ICU patients. The retrospective aspect enabled the investigation of patient records from the onset of the pandemic, offering a comprehensive view of the evolving clinical scenario in Saudi Arabia. Table 1 shows the inclusion and exclusion criteria.

**Table 1:** Inclusion and exclusion criteria COVID-19: Coronavirus disease 2019; BMI: body mass index; ICU: intensive care unit

Criteria	Details
Inclusion	- Adult patients (aged 18+) in Saudi Arabian ICUs - Diagnosed with COVID-19 - Confirmed COVID-19 diagnosis - Documented BMI at ICU admission - Comprehensive electronic health records available for retrospective analysis - Representative of Saudi Arabian ICU population
Exclusion	- Incomplete medical records, especially missing BMI data at ICU admission - Pre-existing conditions affecting BMI or COVID-19 severity (e.g., uncontrolled diabetes, chronic pulmonary diseases) - Patients receiving end-of-life care - Advanced cardiovascular diseases or malignancies

Statistical methods

In our study, we conducted a statistical analysis using IBM SPSS Statistics for Windows, Version 28 (Released 2021; IBM Corp., Armonk, New York, United States). Descriptive statistics were employed to summarize demographic characteristics, presenting counts and percentages for categorical variables such as gender, pregnancy status, and nationality. Mean and standard deviation values were calculated for continuous variables including age and BMI. Chi-square tests were utilized to examine associations between categorical variables, such as gender and pregnancy status. T-tests were employed to compare means for variables like BMI between groups, such as patients with and without microbiological cure. ANOVA was applied to assess the impact of BMI on multiple outcomes, including ICU discharge status and hospital discharge outcomes.

Ethical considerations

Ethical approval was obtained from King Faisal University KFU-REC-2024-JAN-ETHICS1,932. The study adhered to the principles of the Declaration of Helsinki, ensuring patient confidentiality and data privacy at all times. Informed consent was waived due to the retrospective nature of the study, under the condition of strict data anonymization.

Data quality assurance

Robust quality control measures were in place. These included double data entry, random checks for data accuracy, and regular audits. A data monitoring committee was established to oversee the data collection and analysis processes, ensuring adherence to the study protocol and the integrity of the findings.

## Results

Demographic characteristics

As shown in Table 2, the study comprised a total of 1,060 participants, with an average age of 56 years (±15). Gender distribution indicated that 773 participants (72.9%) were male, while 287 (27.1%) were female, with 16 of the female participants reporting pregnancy (5.7%). The mean BMI was 29 (±6). In terms of nationality, 571 participants (53.9%) were Saudi, and 489 (46.1%) were non-Saudi. Most participants were not healthcare workers (94.5%), and a small percentage had traveled outside of Saudi (0.4%). The majority of cases were admitted from home (84.8%).

**Table 2:** Demographic characteristics Demographic characteristics include age, gender, pregnancy status, BMI, healthcare worker status, travel history, and hospital admission sources. BMI: Body mass index

Variables	Options	N	(%)
Age [Years]	Mean ± SD		56 ± 15
Gender			
Female		287	27.1%
Male		773	72.9%
If female, pregnant?			
No		271	94.3%
Yes		16	5.7%
BMI [kg/m2]	Mean ± SD		29 ± 6
Was patient Saudi or non-Saudi?			
Non-Saudi		489	46.1%
Saudi		571	53.9%
Healthcare worker			
No		1002	94.5%
Yes		58	5.5%
Did the case travel outside of Saudi?			
No		1056	99.6%
Yes		4	0.4%
Hospital admission source			
Home		899	84.8%
Nursing home		3	0.3%
Transfer from other facilities		7	0.7%
Other		151	14.2%

Characteristics of the ICU 

As shown in Table 3, the ICU characteristics presented mean values for length of stay (LOS) at 13 days (SD=13), mechanical ventilation (MV) duration at 13 days (SD=11), and hospital LOS at 61 days (SD=1350). The findings offer insights into the critical care experiences of the study population, highlighting the average durations for ICU and hospital stays as well as mechanical ventilation.

**Table 3:** ICU characteristics Intensive care unit (ICU) characteristics encompassing ICU length of stay (LOS), mechanical ventilation duration, and hospital length of stay.

Variables	Mean	SD
ICU LOS (d)	13	13
MV Duration (d)	13	11
Hospital LOS (d)	61	13

Correlation between BMI and ICU characteristics

As shown in Table 4, the correlation analysis between BMI and ICU outcomes showed minimal associations. Specifically, BMI exhibited a negligible negative correlation with ICU LOS (r=-0.003, p=0.933, 95% CI [-0.063, 0.058]), a positive but non-significant correlation with MV duration (r=0.067, p=0.118, 95% CI [-0.017, 0.150]), and a negligible negative correlation with hospital LOS (r=-0.028, p=0.371, 95% CI [-0.088, 0.033]).

**Table 4:** Correlation between BMI and ICU characteristics Pearson correlation coefficients explore associations between BMI and ICU outcomes, including ICU length of stay, mechanical ventilation duration, and hospital LOS. BMI: Body mass index; ICU: intensive care unit; LOS: length of stay

Variables		Pearson Correlation	p-value	95% Confidence Intervals	
				Lower	Upper
BMI	ICU LOS (d)	-0.003	0.933	-0.063	0.058
	MV Duration (d)	0.067	0.118	-0.017	0.150
	Hospital LOS (d)	-0.028	0.371	-0.088	0.033

Outcomes of ICU patients with COVID-19

As shown in Table 5, among them, 16.0% (N=170) achieved microbiological cure, defined as two consecutive negative COVID-19 test samples, while 84.0% (N=890) did not. For patients with eight or more days of ICU stay not ventilated, the majority, 92.5% (N=981), were discharged from the ICU, while 2.1% (N=22) remained in the ICU without ventilation, and 5.4% (N=57) were still in the ICU with ventilation. In terms of ICU discharge outcomes, 37.8% (N=401) resulted in death, 56.0% (N=594) were discharged home, and 6.1% (N=65) transferred to another facility. Hospital discharge outcomes showed that 38.7% (N=410) resulted in death, 55.0% (N=583) were discharged home alive, and 6.3% (N=67) transferred to another facility.

**Table 5:** Outcomes of ICU patients with COVID-19 Outcomes detailing microbiological cure, ICU discharge status, transfer outcomes, and hospital discharge outcomes. COVID-19: Coronavirus disease 2019; ICU: intensive care unit

Variable	Options		N	%
Microbiological cure (defined as two consecutive samples negative COVID Yes9 test)		No	890	84.0%
		Yes	170	16.0%
Still in the ICU, not ventilated 8 days of ICU stay, the patient is		Discharged from ICU	981	92.5%
		Still in ICU, not ventilated	22	2.1%
		Still in ICU, ventilated	57	5.4%
Transfer to another facility ICU discharge outcome		Death	401	37.8%
		Discharge home	594	56.0%
		Transfer to another facility	65	6.1%
Discharge home alive Hospital discharge outcome		Death	410	38.7%
		Discharge home alive	583	55.0%
		Transfer to another facility	67	6.3%

Effect of BMI on outcomes of ICU COVID-19 patients

As shown in Table 6, the analysis of the effect of BMI on ICU outcomes in COVID-19 patients indicated that there were no significant differences in mean BMI between those who achieved microbiological cure and those who did not (29 vs. 30, p=0.235). Similarly, BMI did not significantly differ between patients with different outcomes regarding ICU discharge (p=0.112), transfer to another facility after ICU discharge (p=0.574), and hospital discharge outcomes (p=0.321).

**Table 6:** Effect of BMI on outcomes of ICU COVID-19 patients The impact of BMI on outcomes for ICU COVID-19 patients, employing statistical comparisons with p-values using ANOVA or t-test. BMI: Body mass index; ICU: intensive care unit; COVID-19: coronavirus disease 2019

Variables		BMI		p-value
		Mean	SD	
Microbiological cure (defined as 2 consecutive samples negative COVID Yes9 test)	No	29	6	0.235
	Yes	30	6	
At Still in ICU, not ventilated 8 days of ICU stay, the patient is:	Discharged from ICU	29	6	0.112
	Still in ICU, not ventilated	28	6	
	Still in ICU, ventilated	32	8	
Transfer to another facility ICU discharge outcome	Death	30	6	0.574
	Discharge home	29	6	
	Transfer to another facility	29	5	
Discharge home alive Hospital discharge outcome	Death	30	6	0.321
	Discharge home alive	29	6	
	Transfer to another facility	29	6	

Our study provided a thorough examination of the demographic characteristics, ICU outcomes, and the impact of BMI on patients with COVID-19. The analysis revealed notable percentages of patients achieving microbiological cure and diverse outcomes upon ICU and hospital discharge. Despite employing chi-square, t-tests, and ANOVA, BMI did not emerge as a significant predictor for the investigated outcomes. These findings contribute valuable insights into the clinical profile of COVID-19 patients, emphasizing the multifaceted nature of recovery and the need for nuanced approaches in patient management.

## Discussion

This study, conducted in a Saudi Arabian context, investigated the correlation between BMI and the severity of COVID-19 in ICU patients. Contrary to our initial hypothesis and existing literature [14,15], our findings did not demonstrate a significant association between BMI and COVID-19 severity. This lack of correlation challenges the widely accepted notion that higher BMI is a determinant of increased COVID-19 severity, a trend observed in other regions such as Latin America [16] and general populations [17].

Our retrospective cohort design, which capitalized on a comprehensive analysis of existing patient records, presented a unique opportunity to examine a large dataset. However, this approach brought with it limitations like potential biases in record-keeping and the retrospective nature of data collection, which could affect the interpretation of our findings.

Interestingly, our results diverge from previous studies that have established a clear connection between obesity and heightened vulnerability to viral infections [18], including the specific role of visceral adiposity in exacerbating COVID-19 severity [19,20]. This divergence invites a re-examination of the relationship between BMI and COVID-19 in different demographic contexts. It suggests that factors other than BMI might play more significant roles in the severity of COVID-19 in the Saudi Arabian population.

The broader implications of our study extend to public health policies and interventions. While the global emphasis on managing obesity as part of COVID-19 mitigation strategies remains relevant, our findings highlight the necessity of context-specific approaches, especially in regions like Saudi Arabia. This aligns with the recommendations from studies emphasizing the impact of obesity on respiratory diseases [21] and influenza [22,23].

Nevertheless, the limitations of our study, including its retrospective design and the specific demographic characteristics of the Saudi Arabian health system, must be acknowledged. These factors limit the generalizability of our findings and preclude the establishment of causal relationships.

Future research should, therefore, aim to conduct prospective studies that can more accurately establish causality between BMI and COVID-19 severity. Additionally, investigating the effectiveness of targeted BMI reduction interventions in COVID-19 patients could provide valuable insights.

Our study offered a unique perspective by exploring the association between BMI and COVID-19 severity within the Saudi Arabian context. It challenges existing assumptions and underscores the importance of a nuanced approach in both clinical management and public health policy formulation, especially in populations with distinct demographic and health profiles. Our findings contribute to the global dialogue on COVID-19, emphasizing the need for region-specific research and interventions.

## Conclusions

Our study in Saudi Arabia revealed no significant correlation between BMI and COVID-19 severity in ICU patients, challenging the widely accepted global trends. This finding underscores the importance of considering regional differences in COVID-19 research and response strategies. Although limited by its retrospective design and regional focus, our study highlights the need for context-specific health policies and future prospective research. It shifts the narrative on the relationship between obesity and COVID-19, suggesting that other factors might be more influential in certain populations, and emphasizes the importance of tailored approaches in managing the pandemic in diverse demographic settings.

## References

[1] AlBahrani S, Al-Maqati TN, Al Naam YA (2023). The association of body mass index with COVID-19 complications and survival rate at a tertiary hospital. Life (Basel).

[2] Gao M, Piernas C, Astbury NM, Hippisley-Cox J, O'Rahilly S, Aveyard P, Jebb SA (2021). Associations between body-mass index and COVID-19 severity in 6·9 million people in England: a prospective, community-based, cohort study. Lancet Diabetes Endocrinol.

[3] Jayanama K, Srichatrapimuk S, Thammavaranucupt K (2021). The association between body mass index and severity of Coronavirus Disease 2019 (COVID-19): a cohort study. PLoS One.

[4] Almuqbil M, Almoteer AI, Suwayyid AM (2023). Characteristics of COVID-19 patients admitted to intensive care Unit in Multispecialty Hospital of Riyadh, Saudi Arabia: a retrospective study. Healthcare (Basel).

[5] Townsend MJ, Kyle TK, Stanford FC (2020). Outcomes of COVID-19: disparities in obesity and by ethnicity/race. Int J Obes (Lond).

[6] Hippisley-Cox J, Young D, Coupland C (2020). Risk of severe COVID-19 disease with ACE inhibitors and angiotensin receptor blockers: cohort study including 8.3 million people. Heart.

[7] Sattar N, Ho FK, Gill JM (2020). BMI and future risk for COVID-19 infection and death across sex, age and ethnicity: preliminary findings from UK biobank. Diabetes Metab Syndr.

[8] Hewitt J, Carter B, Vilches-Moraga A (2020). The effect of frailty on survival in patients with COVID-19 (COPE): a multicentre, European, observational cohort study. Lancet Public Health.

[9] Rietman ML, van der A DL, van Oostrom SH (2018). The association between BMI and different frailty domains: a U-shaped curve?. J Nutr Health Aging.

[10] Lockhart SM, O'Rahilly S (2020). When two pandemics meet: why is obesity associated with increased COVID-19 mortality?. Med.

[11] Dalamaga M, Christodoulatos GS, Karampela I, Vallianou N, Apovian CM (2021). Understanding the co-epidemic of obesity and COVID-19: current evidence, comparison with previous epidemics, mechanisms, and preventive and therapeutic perspectives. Curr Obes Rep.

[12] Michalakis K, Panagiotou G, Ilias I, Pazaitou-Panayiotou K (2021). Obesity and COVID-19: a jigsaw puzzle with still missing pieces. Clin Obes.

[13] Pinhel MA, Watanabe LM, Noronha NY, Junior WS, Nonino CB (2021). The intersection between COVID-19 and obesity in the context of an emerging country. Clin Nutr ESPEN.

[14] Halpern B, Louzada ML, Aschner P (2021). Obesity and COVID-19 in Latin America: a tragedy of two pandemics-official document of the Latin American Federation of Obesity Societies. Obes Rev.

[15] Lavie CJ, Coursin DB, Long MT (2021). The obesity paradox in infections and implications for COVID-19. Mayo Clin Proc.

[16] Karampela I, Vallianou N, Magkos F, Apovian CM, Dalamaga M (2022). Obesity, hypovitaminosis D, and COVID-19: the Bermuda Triangle in public health. Curr Obes Rep.

[17] Gonçalves TJ, Gonçalves SE, Guarnieri A (2020). Prevalence of obesity and hypovitaminosis D in elderly with severe acute respiratory syndrome coronavirus 2 (SARS-CoV-2). Clin Nutr ESPEN.

[18] Battisti S, Pedone C, Napoli N (2020). Computed tomography highlights increased visceral adiposity associated with critical illness in COVID-19. Diabetes Care.

[19] Watanabe M, Caruso D, Tuccinardi D (2020). Visceral fat shows the strongest association with the need of intensive care in patients with COVID-19. Metabolism.

[20] Chandarana H, Dane B, Mikheev A, Taffel MT, Feng Y, Rusinek H (2021). Visceral adipose tissue in patients with COVID-19: risk stratification for severity. Abdom Radiol (NY).

[21] Umbrello M, Fumagalli J, Pesenti A, Chiumello D (2019). Pathophysiology and management of acute respiratory distress syndrome in obese patients. Semin Respir Crit Care Med.

[22] Braun ES, Crawford FW, Desai MM (2015). Obesity not associated with severity among hospitalized adults with seasonal influenza virus infection. Infection.

[23] Ni YN, Luo J, Yu H (2017). Can body mass index predict clinical outcomes for patients with acute lung injury/acute respiratory distress syndrome? A meta-analysis. Crit Care.

